# Supplementary material for: Tracking down carbon inputs underground from an arid zone Australian calcrete
Source: PLoS One. 2020 Aug 28;15(8):e0237730. doi: 10.1371/journal.pone.0237730 (PMC7454941; doi:10.1371/journal.pone.0237730)
Supplement: S1 Table — BP: before present with present being 1950 AD; pMC: percent of modern carbon. (DOCX) [file pone.0237730.s001.docx]

**S1 Table**. DOC (Dissolved Organic Carbon) and DIC (Dissolved Organic Carbon) concentrations (mg/L), δ^13^C DOC, δ^13^C DIC, pMC DOC, Δ^14^C DOC, Conventional Age DOC, pMC DIC, Δ^14^C DIC, Conventional Age DIC for the bores W4 and D13. BP: before present with present being 1950 AD; pMC: percent of modern carbon.
