# Supplementary material for: Tracking down carbon inputs underground from an arid zone Australian calcrete
Source: PLoS One. 2020 Aug 28;15(8):e0237730. doi: 10.1371/journal.pone.0237730 (PMC7454941; doi:10.1371/journal.pone.0237730)
Supplement: S2 Table — Adapted from Coble et al. [111]. Note: em = emission wavelengths, ex = excitation wavelengths. (DOCX) [file pone.0237730.s002.docx]

**S2 Table**. Fluorescence/absorbance indices and their definitions. Adapted from Coble et al. [111]. Note: em = emission wavelengths, ex = excitation wavelengths.

| **Fluorescence /absorbance index** | **Parameters/description** | **Interpretation** | **Reference** |
| --- | --- | --- | --- |
|  |  |  |  |
| Humification Index  (HIX_EM_) | At ex 254 nm, area of peak under em 435–480 nm divided by area under em  300–345 nm + 435–480 nm. | Higher numbers are indicative of lower H:C ratios, attributed to a greater degree of humification. | [75,77] |
| Freshness Index  (BIX) | Intensity at em 380 nm divided by  max intensity between em 420 nm and em 435 nm at ex  310 nm. | Indicates proportion of recently produced DOM. | [79,112] |
| Fluorescence Index  (FI) | The ratio of em 450 nm and em 500 nm at ex 370 nm. | Indicates if precursor material for DOM is of a more microbial (FI ~ 1.8) in nature or more terrestrially derived (FI ~1.2). | [77] |
| Spectral Slope Ratio  (SR) | The ratio of S275-295 to S350-400. | Lower S275-295 correlated with higher aromatic content and higher molecular weight, i.e. if SR is above 1 then the CDOM is more marine-like. | [81] |
| Coble peaks  (A, B, C, M, T) | A: Humic-like ex = 250–260 nm, em = 380–480 nm. B: Tyrosine-like ex = 270–280 nm, em = 300–320 nm. C: Humic-like ex = 330–350 nm, em = 420–480 nm. M: Marine humic-like ex = 310–320 nm, em = 380–420 nm. T: Tryptophan-like ex = 270–280 nm, em = 320–350 nm. | Identifies the intensity of protein-like and/or humic-like peaks. | [113] |
| C:A | The ratio of Peak C to Peak A intensity. | An indication of the amount of humic-like vs. fulvic-like fluorescence in a sample. | [25,113] |
| C:M | The ratio of Peak C to Peak M intensity. | An indication of the amount of diagenetically altered (blue- shifted) fluorescence in a sample. | [82,113] |
| SUVA_254_ | Absorption coefficient at 254 nm divided by DOC concentration. | Absorbance per unit carbon. Typically a higher number is associated with greater aromatic content | [114] |
|  |  |  |  |

**Additional references**

1. Coble PG, Lead J, Baker A, Reynolds DM, Spencer RG, editors. Aquatic organic matter fluorescence. Cambridge University Press; 2014 Jul 14.
2. Huguet A, Vacher L, Relexans S, Saubusse S, Froidefond JM, Parlanti E. Properties of fluorescent dissolved organic matter in the Gironde Estuary. Organic Geochemistry. 2009 Jun 1;40(6):706-19.
3. Coble PG. Characterization of marine and terrestrial DOM in seawater using excitation-emission matrix spectroscopy. Marine chemistry. 1996 Jan 1;51(4):325-46.
4. Weishaar J, Aiken G, Fram MS, Bergamaschi BA, Fujii R. Evaluation of specific UV absorbance as an indicator of the chemical content of dissolved organic carbon. In ‘Abstracts of papers of the American Chemical society’. 2001 Aug 1;222:451-451.
