# Supplementary material for: Tracking down carbon inputs underground from an arid zone Australian calcrete
Source: PLoS One. 2020 Aug 28;15(8):e0237730. doi: 10.1371/journal.pone.0237730 (PMC7454941; doi:10.1371/journal.pone.0237730)
Supplement: S3 Table — Pathways in bold indicate the significantly (P < 0.05) overrepresented pathways in one of the two rainfall periods. (DOCX) [file pone.0237730.s003.docx]

**S3 Table**. Abundances of PICRUSt2 outputs relating to carbon metabolism (KO, level 3) and degradative pathways (MetaCyc). Pathways in bold indicate the significantly (*P* < 0.05) overrepresented pathways in one of the two rainfall periods.

| **Carbon metabolism - Kegg** | | | | | | |
| --- | --- | --- | --- | --- | --- | --- |
| **Pathway** | **HR_1** | **HR_2** | **HR_3** | **LR_1** | **LR_2** | **LR_3** |
| Glycolysis | 7475 | 6729 | 7102 | 10596 | 12413 | 13758 |
| **Pentose phosphate pathway** | 3345 | 2940 | 3142 | 4040 | 4867 | 4303 |
| Citrate cycle | 5124 | 3854 | 4489 | 7233 | 9925 | 8574 |
| Methane metabolism | 3660 | 3036 | 3348 | 3713 | 4661 | 6245 |
| **Reductive pentose phosphate cycle** | 3285 | 2791 | 3038 | 3255 | 3983 | 4206 |
| Reductive citrate cycle | 4498 | 4799 | 4649 | 7553 | 10444 | 10894 |
| **Reductive acetyl-CoA pathway** | 1137 | 980 | 1058 | 1093 | 1556 | 861 |
| 3-Hydroxypropionate bi-cycle | 3259 | 2391 | 2825 | 3760 | 5563 | 3017 |
| Hydroxypropionate-hydroxybutylate cycle | 1675 | 497 | 1086 | 1848 | 2738 | 1597 |
| **Dicarboxylate-hydroxybutyrate cycle** | 3831 | 3092 | 3462 | 6206 | 8896 | 9555 |
|  |  |  |  |  |  |  |
|  |  |  |  |  |  |  |
| **Degradative pathways - MetaCyc** | | | | | | |
| **pathway** | **HR_1** | **HR_2** | **HR_3** | **LR_1** | **LR_2** | **LR_3** |
| 3-phenylpropanoate and 3-(3-hydroxyphenyl)propanoate degradation | 0 | 0 | 76 | 15 | 50 | 12 |
| 3-phenylpropanoate and 3-(3-hydroxyphenyl)propanoate degradation to 2-oxopent-4-enoate | 0 | 0 | 41 | 7 | 29 | 4 |
| 3-phenylpropanoate degradation | 1 | 28 | 1 | 0 | 0 | 0 |
| 4-aminobutanoate degradation V | 67 | 61 | 169 | 115 | 69 | 236 |
| 4-deoxy-L-threo-hex-4-enopyranuronate degradation | 1 | 76 | 98 | 0 | 0 | 0 |
| 4-hydroxyphenylacetate degradation | 4 | 41 | 38 | 8 | 6 | 22 |
| **4-methylcatechol degradation (ortho cleavage)** | 55 | 35 | 87 | 24 | 1 | 2 |
| acetylene degradation | 50 | 175 | 246 | 108 | 139 | 273 |
| adenosine nucleotides degradation II | 414 | 24 | 315 | 343 | 509 | 231 |
| allantoin degradation IV (anaerobic) | 0 | 0 | 0 | 28 | 2 | 0 |
| allantoin degradation to glyoxylate III | 72 | 5 | 35 | 59 | 4 | 4 |
| aromatic biogenic amine degradation (bacteria) | 0 | 0 | 82 | 17 | 55 | 23 |
| **Aromatic compounds degradation via beta-ketoadipate** | 93 | 35 | 88 | 25 | 1 | 3 |
| catechol degradation I (meta-cleavage pathway) | 0 | 0 | 105 | 25 | 67 | 29 |
| **Catechol degradation III (ortho-cleavage pathway)** | 93 | 35 | 88 | 25 | 1 | 3 |
| **Catechol degradation to beta-ketoadipate** | 87 | 32 | 92 | 24 | 2 | 15 |
| chlorosalicylate degradation | 0 | 0 | 0 | 3 | 2 | 12 |
| cinnamate and 3-hydroxycinnamate degradation to 2-oxopent-4-enoate | 0 | 0 | 41 | 7 | 29 | 4 |
| creatinine degradation I | 266 | 2 | 24 | 12 | 3 | 0 |
| D-fructuronate degradation | 42 | 111 | 222 | 50 | 86 | 156 |
| D-galactarate degradation I | 3 | 32 | 26 | 4 | 3 | 19 |
| D-galacturonate degradation I | 3 | 51 | 114 | 8 | 4 | 124 |
| D-glucarate degradation I | 3 | 33 | 27 | 5 | 4 | 32 |
| fucose degradation | 2 | 44 | 23 | 9 | 11 | 3 |
| galactose degradation I (Leloir pathway) | 121 | 223 | 373 | 177 | 136 | 360 |
| gallate degradation I | 0 | 0 | 0 | 5 | 3 | 12 |
| gallate degradation II | 0 | 0 | 0 | 5 | 3 | 12 |
| **Glucose and glucose-1-phosphate degradation** | 55 | 60 | 148 | 36 | 5 | 24 |
| glycine betaine degradation I | 168 | 0 | 15 | 10 | 5 | 0 |
| **Glycogen degradation I (bacterial)** | 89 | 207 | 249 | 269 | 390 | 379 |
| guanosine nucleotides degradation III | 397 | 64 | 321 | 338 | 506 | 246 |
| L-1,2-propanediol degradation | 0 | 2 | 52 | 145 | 37 | 149 |
| lactose and galactose degradation I | 34 | 101 | 110 | 5 | 53 | 59 |
| **L-arginine degradation II (AST pathway)** | 90 | 63 | 102 | 20 | 5 | 27 |
| L-histidine degradation I | 112 | 54 | 242 | 65 | 19 | 67 |
| L-histidine degradation II | 1 | 8 | 27 | 6 | 5 | 23 |
| L-leucine degradation I | 324 | 3 | 204 | 154 | 210 | 163 |
| L-rhamnose degradation I | 27 | 42 | 115 | 34 | 29 | 22 |
| L-tryptophan degradation to 2-amino-3-carboxymuconate semialdehyde | 0 | 0 | 72 | 0 | 0 | 0 |
| L-tyrosine degradation I | 108 | 3 | 181 | 30 | 10 | 19 |
| mannan degradation | 0 | 0 | 175 | 0 | 0 | 0 |
| methylgallate degradation | 0 | 0 | 0 | 6 | 3 | 15 |
| methylphosphonate degradation I | 172 | 47 | 127 | 27 | 41 | 18 |
| myo-, chiro- and scillo-inositol degradation | 10 | 41 | 89 | 71 | 42 | 72 |
| myo-inositol degradation I | 4 | 35 | 65 | 58 | 38 | 39 |
| nicotinate degradation I | 1 | 4 | 15 | 3 | 3 | 14 |
| phenylacetate degradation I (aerobic) | 65 | 44 | 46 | 26 | 1 | 4 |
| protocatechuate degradation II (ortho-cleavage pathway) | 206 | 50 | 201 | 57 | 16 | 59 |
| **Purine nucleobases degradation I (anaerobic)** | 130 | 50 | 140 | 12 | 8 | 0 |
| purine nucleotides degradation II (aerobic) | 380 | 44 | 371 | 404 | 583 | 185 |
| purine ribonucleosides degradation | 254 | 181 | 169 | 136 | 114 | 177 |
| starch degradation V | 85 | 183 | 229 | 217 | 135 | 333 |
| sucrose degradation III (sucrose invertase) | 53 | 182 | 237 | 57 | 89 | 309 |
| sucrose degradation IV (sucrose phosphorylase) | 53 | 58 | 133 | 81 | 76 | 164 |
| superpathway of &beta;-D-glucuronide and D-glucuronate degradation | 31 | 81 | 109 | 12 | 6 | 126 |
| superpathway of aerobic toluene degradation | 0 | 0 | 24 | 15 | 1 | 4 |
| superpathway of D-glucarate and D-galactarate degradation | 3 | 32 | 26 | 4 | 3 | 19 |
| superpathway of glucose and xylose degradation | 125 | 180 | 308 | 225 | 101 | 128 |
| superpathway of hexitol degradation (bacteria) | 87 | 163 | 209 | 30 | 137 | 146 |
| superpathway of hexuronide and hexuronate degradation | 3 | 51 | 96 | 7 | 1 | 110 |
| superpathway of L-arginine and L-ornithine degradation | 5 | 54 | 48 | 9 | 8 | 36 |
| superpathway of L-arginine, putrescine, and 4-aminobutanoate degradation | 5 | 54 | 48 | 9 | 8 | 36 |
| superpathway of N-acetylglucosamine, N-acetylmannosamine and N-acetylneuraminate degradation | 7 | 75 | 55 | 10 | 45 | 138 |
| superpathway of N-acetylneuraminate degradation | 21 | 151 | 131 | 31 | 100 | 230 |
| **Superpathway of ornithine degradation** | 72 | 48 | 65 | 29 | 18 | 23 |
| superpathway of phenylethylamine degradation | 62 | 43 | 30 | 30 | 1 | 5 |
| superpathway of purine deoxyribonucleosides degradation | 230 | 149 | 230 | 128 | 171 | 187 |
| superpathway of pyrimidine deoxyribonucleosides degradation | 55 | 120 | 167 | 73 | 67 | 195 |
| **Superpathway of salicylate degradation** | 100 | 34 | 85 | 23 | 1 | 3 |
| superpathway of taurine degradation | 13 | 0 | 6 | 0 | 2 | 0 |
| toluene degradation I (aerobic) (via o-cresol) | 0 | 0 | 107 | 28 | 69 | 32 |
| toluene degradation II (aerobic) (via 4-methylcatechol) | 0 | 0 | 107 | 28 | 69 | 32 |
| toluene degradation III (aerobic) (via p-cresol) | 66 | 41 | 60 | 32 | 1 | 4 |
| toluene degradation IV (aerobic) (via catechol) | 0 | 1 | 60 | 12 | 2 | 6 |
| urate biosynthesis/inosine 5'-phosphate degradation | 478 | 395 | 478 | 584 | 767 | 361 |
| vitamin B6 degradation | 0 | 1 | 9 | 27 | 6 | 2 |
