# Supplementary material for: Tracking down carbon inputs underground from an arid zone Australian calcrete
Source: PLoS One. 2020 Aug 28;15(8):e0237730. doi: 10.1371/journal.pone.0237730 (PMC7454941; doi:10.1371/journal.pone.0237730)
Supplement: S4 Table — Na: Not available. Units of δ18O and 2H in per mil (‰), and units of tritium in TU (Tritium Units). (DOCX) [file pone.0237730.s004.docx]

**S4 Table**. Hydrochemical values of the bores D13 and W4 under LR and HR. Na: Not available. Units of δ^18^O and ^2^H in per mil (‰), and units of tritium in TU (Tritium Units).
