# Supplementary material for: Tracking down carbon inputs underground from an arid zone Australian calcrete
Source: PLoS One. 2020 Aug 28;15(8):e0237730. doi: 10.1371/journal.pone.0237730 (PMC7454941; doi:10.1371/journal.pone.0237730)
Supplement: S1 Fig — Two-dimensional (left panel) and three-dimensional (mid panel) fluorescence landscapes, and the excitation (grey line) and emission (black line) spectra (right panel) for the five different components identified by the PARAFAC model. Intensity is scaled to a maximum fluorescence of 1. (DOCX) [file pone.0237730.s005.docx]

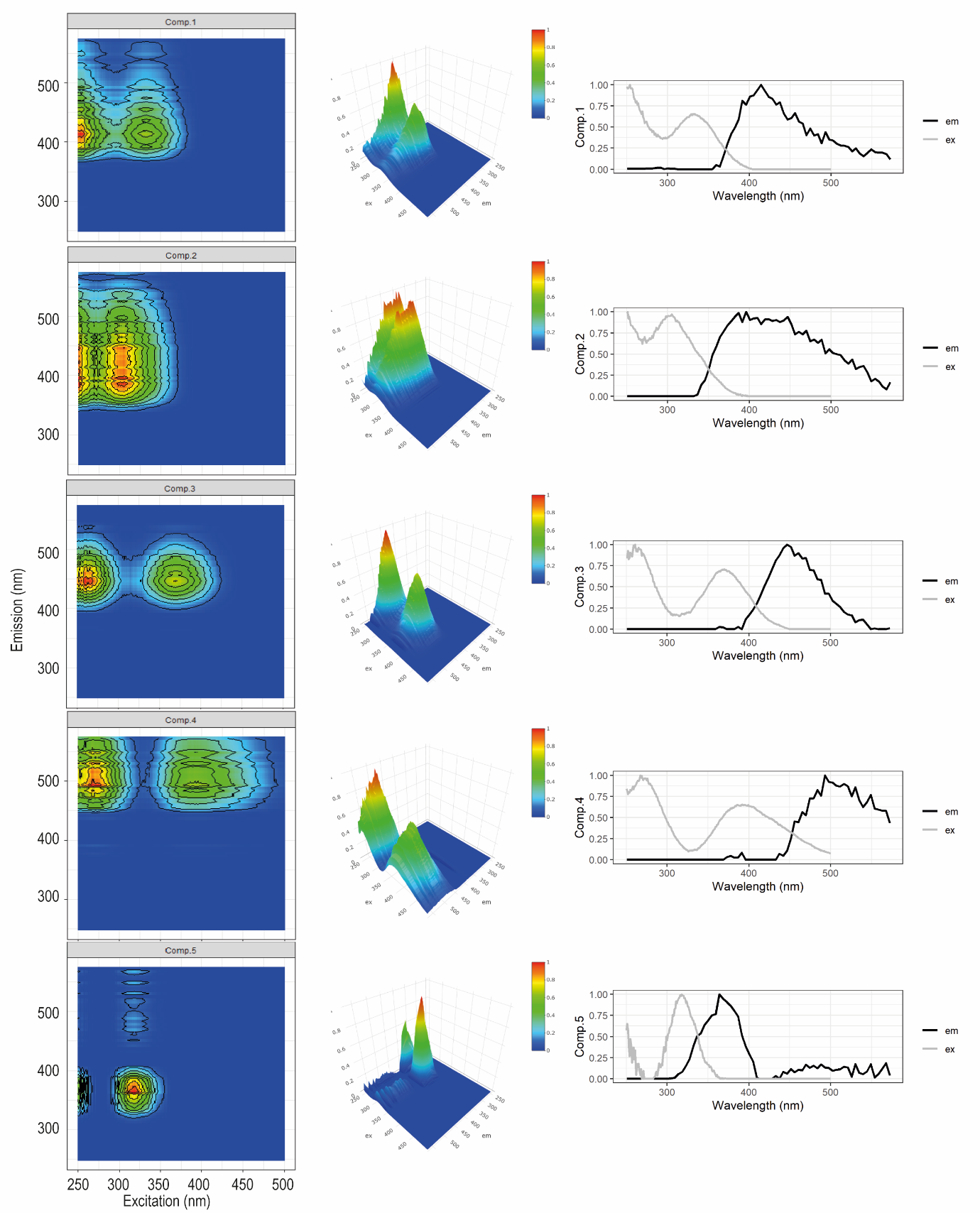


**S1 Fig**. Two-dimensional (left panel) and three-dimensional (mid panel) fluorescence landscapes, and the excitation (grey line) and emission (black line) spectra (right panel) for the five different components identified by the PARAFAC model. Intensity is scaled to a maximum fluorescence of 1.
