# Supplementary material for: Tracking down carbon inputs underground from an arid zone Australian calcrete
Source: PLoS One. 2020 Aug 28;15(8):e0237730. doi: 10.1371/journal.pone.0237730 (PMC7454941; doi:10.1371/journal.pone.0237730)
Supplement: S2 Fig — Abundances corresponding to the 37 ZOTUs without a reference/belonging to uncultured bacterium were removed from the figure for clarity purposes. (DOCX) [file pone.0237730.s006.docx]

**
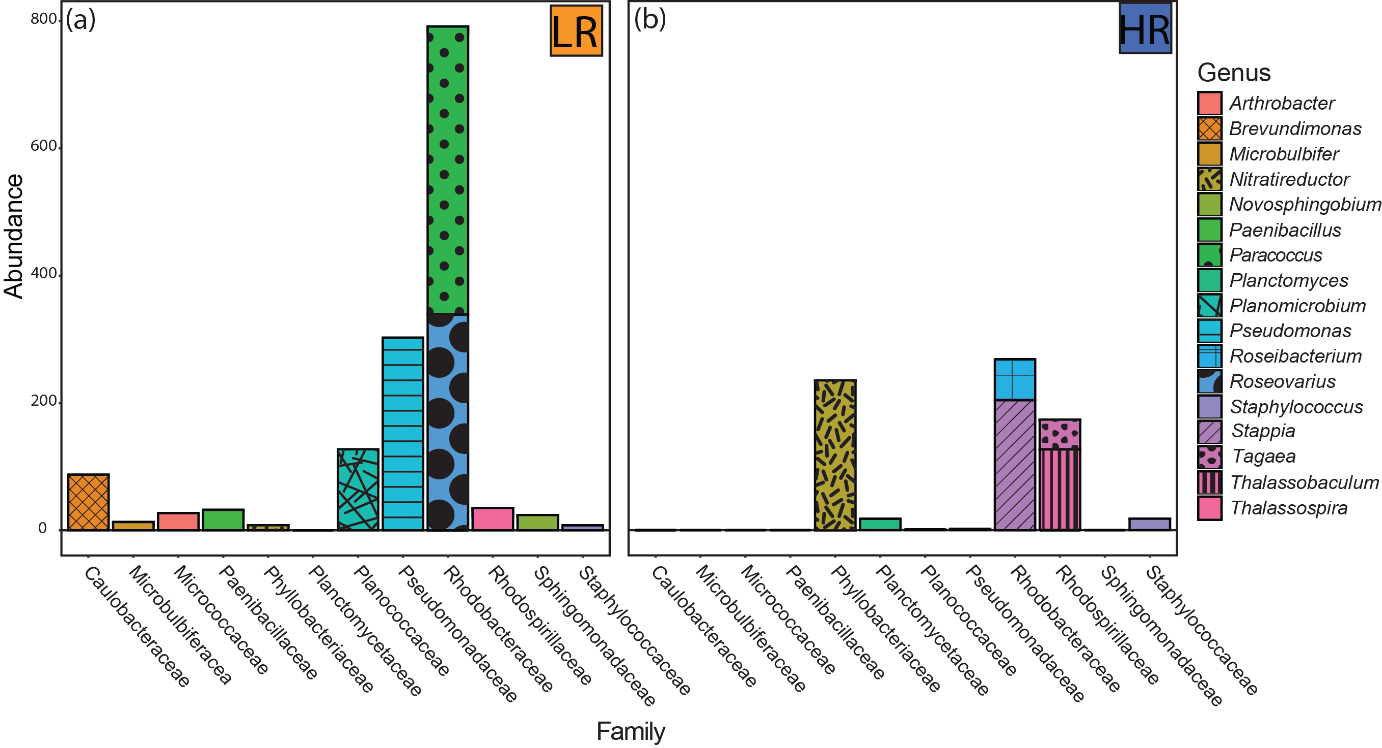
**

**S2 Fig.** Bar plots illustrating the abundances of genus and families under LR and HR. Abundances corresponding to the 37 ZOTUs without a reference/belonging to uncultured bacterium were removed from the figure for clarity purposes.
